# Supplementary material for: Are long-term growth responses to elevated pCO2 sex-specific in fish?
Source: PLoS One. 2020 Jul 17;15(7):e0235817. doi: 10.1371/journal.pone.0235817 (PMC7367484; doi:10.1371/journal.pone.0235817)
Supplement: S1 Table — The number and length of Spawning ripe M. menidia used to fertilize trials 1–4. (DOCX) [file pone.0235817.s002.docx]

**Table S1. The number and length of Spawning ripe *M. menidia* used to fertilize trials 1-4.**

| **Collection date** | **Fertilization date** | **# Female spawners** | **Female TL** | **# Male spawners** | **Male TL** |
| --- | --- | --- | --- | --- | --- |
| 5/1/2015 | 5/3/2015 | 28 | 9.8 ± 1.0 | 21 | 9.5 ± 1.0 |
| 5/3/2016 | 5/4/2016 | 25 | 9.0 ± 1.6 | 63 | 8.1 ± 1.0 |
| 5/18/2016 | 5/19/2016 | 32 | 10.3 ±1.3 | 40 | 8.7 ± 1.1 |
| 6/28/2017 | 6/29/2017 | 12 | 10.0 ± 1.0 | 15 | 9.2 ± 1.3 |

Spawning adults were collected from Mumford Cove, CT. Total lengths of adults used for each fertilization event as shown as mean total lengths (TL, cm) ± standard deviation.
