# Supplementary material for: Are long-term growth responses to elevated pCO2 sex-specific in fish?
Source: PLoS One. 2020 Jul 17;15(7):e0235817. doi: 10.1371/journal.pone.0235817 (PMC7367484; doi:10.1371/journal.pone.0235817)
Supplement: S2 Table — M. menidia. Mean (±s.d.) TL and samples sizes (N) of subsampled offspring from trials 1–4. (DOCX) [file pone.0235817.s003.docx]

**Table S2. *M. menidia.* Mean (±s.d.) TL and samples sizes (N) of subsampled offspring from trials 1-4.**

| **Trial** | **Temp (°C)** | **Age (dph)** | ***p*CO_2_ (µatm)** | **Tank** | **N** | **TL (mm)** |
| --- | --- | --- | --- | --- | --- | --- |
| 1 | 17° | 16 | 450 | 1 | 19 | 9.2 ± 1.3 |
|  |  |  |  | 2 | 18 | 8.6 ± 1.1 |
|  |  |  | 2,200 | 3 | 16 | 8.4 ± 1 |
|  |  |  |  | 4 | 17 | 8.7 ± 1.1 |
|  |  | 36 | 450 | 1 | 10 | 13.4 ± 2.1 |
|  |  |  |  | 2 | 10 | 13.6 ± 2.3 |
|  |  |  | 2,200 | 3 | 10 | 13.6 ± 2.4 |
|  |  |  |  | 4 | 10 | 14.2 ± 2.7 |
|  |  | 68 | 450 | 1 | 10 | 24 ± 2 |
|  |  |  |  | 2 | 10 | 22.6 ± 2.3 |
|  |  |  | 2,200 | 3 | 10 | 22.9 ± 2.9 |
|  |  |  |  | 4 | 10 | 19.7 ± 3.9 |
|  |  | 100 | 450 | 1 | 20 | 30.1 ± 6.3 |
|  |  |  |  | 2 | 8 | 27.2 ± 6.2 |
|  |  |  | 2,200 | 3 | 12 | 25.9 ± 5.2 |
|  |  |  |  | 4 | 16 | 25.3 ± 6.7 |
| 2 | 17° | 21 | 450 | 5 | 30 | 10.1 ± 1.1 |
|  |  |  |  | 6 | 29 | 9.8 ± 1 |
|  |  |  | 2,200 | 7 | 30 | 9.7 ± 0.8 |
|  |  |  |  | 8 | 30 | 9.5 ± 1 |
|  |  | 69 | 450 | 5 | 15 | 26.3 ± 3.9 |
|  |  |  |  | 6 | 15 | 25.3 ± 4 |
|  |  |  | 2,200 | 7 | 15 | 23.7 ± 2.6 |
|  |  |  |  | 8 | 15 | 23.5 ± 3.3 |
|  |  | 103 | 450 | 5 | 16 | 44.8 ± 5.6 |
|  |  |  |  | 6 | 15 | 44.4 ± 6 |
|  |  |  | 2,200 | 7 | 15 | 36.9 ± 9.3 |
|  |  |  |  | 8 | 15 | 40.1 ± 6.4 |
| 3 | 24° | 10 | 450 | 9 | 30 | 9.1 ± 1.3 |
|  |  |  |  | 10 | 30 | 8.8 ± 1.2 |
|  |  |  | 2,200 | 11 | 30 | 8.3 ± 1.3 |
|  |  |  |  | 12 | 30 | 8.1 ± 1.1 |
|  |  | 50 | 450 | 9 | 15 | 29.3 ± 4.1 |
|  |  |  |  | 10 | 15 | 30.7 ± 2.9 |
|  |  |  | 2,200 | 11 | 15 | 26.8 ± 3.8 |
|  |  |  |  | 12 | 15 | 29.7 ± 3.2 |
| 4 | 24° | 35 | 450 | 13 | 36 | 24 ± 2.6 |
|  |  |  | 2200 | 14 | 36 | 24 ± 2.6 |
|  |  | 58 | 450 | 13 | 36 | 34.7 ± 4.2 |
|  |  |  | 2200 | 14 | 36 | 34.1 ± 4.9 |
|  |  | 83 | 450 | 13 | 11 | 48.7 ± 4.1 |
|  |  |  | 2200 | 14 | 12 | 49.6 ± 6.5 |
|  | 28° | 50 | 450 | 15 | 36 | 32.9 ± 3.9 |
|  |  |  | 2200 | 16 | 36 | 32.1 ± 3.7 |
|  |  | 64 | 450 | 15 | 15 | 41.2 ± 2.9 |
|  |  |  | 2200 | 16 | 15 | 40.2 ± 4.2 |
|  |  | 74 | 450 | 15 | 14 | 44.3 ± 3.8 |
|  |  |  | 2200 | 16 | 15 | 42.8 ± 5.4 |
