# Supplementary material for: Are long-term growth responses to elevated pCO2 sex-specific in fish?
Source: PLoS One. 2020 Jul 17;15(7):e0235817. doi: 10.1371/journal.pone.0235817 (PMC7367484; doi:10.1371/journal.pone.0235817)
Supplement: Attachment — Submitted filename: Murray & Baumann - Reviewer responses.docx [file pone.0235817.s005.docx]

PONE-D-19-33606 – Murray & Baumann

**Are long-term growth responses to elevated *p*CO_2_ sex-specific in fish?**

**Reply to editor and reviewer comments**

Dear Dr. Frank Melzner,

We wish to sincerely thank you and both anonymous reviewers for your constructive assessment of our manuscript. As you will see from our point-by-point responses below, we have thoroughly considered each issue, incorporated the reviewer’s suggestions to the best of our abilities and as a result feel that text, presentation and methods have substantially strengthened.

Please find our responses (**bold**) to each reviewer comment (*italics*) below.

**Reviewer 1**

*R1-1:* *The manuscript ‘Are temperature-specific, long-term growth responses to elevated CO2 sex-specific in fish?’ proposes to undertake what is unquestionably an area of significant importance within the biological literature, namely the lack of sex-specific responses being considered in a wide range of fields, and in this case specifically the field of climate change. This topic is also incredibly timely with a number of manuscripts starting to address this topic. Unfortunately however, I feel there are a number of significant methodological flaws with this study, which prevent it being possible to recommend for publication in its current form. Below I outline the major concerns, which will hopefully aid the authors in publishing their data, which definitely has value for the ocean acidification field, but certainly not in the way it is currently presented.*

**We are grateful for reviewer 1’s thorough assessment of our study and for her/his helpful recommendations. As detailed below, we have made substantial revisions to the manuscript based on her/his comments and believe that we have addressed all concerns.**

*R1-2: My first major concern is the presentation of this manuscript as testing sex-specific responses across a range of temperature and pCO2 treatments. The title alludes to this fact, and then the introduction is almost entirely focused on the importance and implications of sex disaggregated results. However, in reality the sex-specific responses in this manuscript are restricted to one temperature treatment (17 degrees). The authors argue this is because temperature has a confounding effect on sex ratio at 24 degrees, preventing comparison. However, sex ratio is only presented for one of the two trials in which organisms were exposed to 24 degrees, and completely omitted from the 28 degree treatment. Was it not measured? Equally, whilst organismal numbers are certainly unbalanced at 24 and 28 degrees, the sex-specific response is still surely valid? As in nature where elevated temperatures and elevated pCO2s co-exist, this same male biased sex ratios would surely be present, and thus the possibility of subsequent sex-specific responses even more important for the future viability of this species, especially if any female enhanced sensitivity is noted. Also clearly this male bias with sex ratio is a major concern under climate change scenarios for this species, a point entirely overlooked in this paper. The alignment of this paper as testing sex-specific responses is not founded, and in reality can only be presented IF the 17 degree data is presented alone, hence removing the multi-stressor angle of the paper, due to the omission of meaningful sex disaggregated data from 2 of the 3 temperature treatments.*

**We were initially reluctant to analyze sex-specific growth patterns from trial 3 due to the large sample size difference between sexes. However, we did sex all trial 3 juveniles to determine sex ratios and do have sex-specific growth data. While the female sample size is low (15-22 females/tank) we are now following Reviewer 1’s recommendation to test for sex-specific effects. Unfortunately, we did not sex juveniles from trial 4 and thus cannot include those data in this analysis. Please refer to comment R1-3 for a comprehensive description of the modified statistical approach.**

**Furthermore, we appreciate R1’s prescience in recognizing the potential demographic effects of warming on silverside populations. This is certainly a topic our research group has pondered. Long-term warming combined with frequent and intense heat waves could severely male-bias the sex ratio of spawning silverside populations. However, the existence of a latitudinal cline in decreasing temperature-dependent sex determination from south to north complicates this prediction. Because southern and warm-adapted genotypes will likely shift northward with future warming, the phenotypic effects of warming on spawning-stock sex ratios may be offset by the underlying genotypic changes. Undoubtedly, a northward shift of southern genotypes will have broad ecological implications (i.e., through changes in population growth rate), but to speculate on this was outside the scope of this study.**

*R1-3: The second major concern is the authors propose to undertake a long-term multi factorial experiment over three years. Whilst this statement is true, it implies a well-designed, replicated fully factorial multi stressor experiment. In reality each trial, of which there were 4, tested the impact of pCO2 on fish growth under a single temperature, in different years. This approach is not valid. The experimental results obtained for the impact of elevated pCO2 on fish growth at 28 degrees, collected in 2017, are not directly comparable to the data collected in 2015 at 17 degrees. There are clearly confounding impacts that invalidate direct statistical comparison. Equally, the authors themselves claim variability in data collected in subsequent years may be due to an optimisation of experimental methodology during subsequent years. So they themselves appreciate that it is impossible to replicate the experiment in subsequent years identically, so designing the experiment to have each treatment tested in different years is a major concern. Data can certainly be compared within years/treatments, but not across treatments as presented.*

**We thank Reviewer 1 for their thoughtful and constructive feedback to improve our analyses of sex-specific growth effects. As we now prominently state in the abstract, long-term experiments on fish are very space demanding, which commonly results in limitations on lab space and logistical capacity. In our case, this precluded completing a fully-factorial 2 CO_2_ × 3 temperatures trial during each experimental year. Despite best efforts to replicate experimental conditions across trials, we agree that small methodological improvements and potential intrinsic differences between fertilization events limit the reliability of a single statistical test to cover all trials. Following R1’s suggestion, we have therefore restructured our analysis to only compare sex-specific CO_2_ effects on growth (TL, wW, and *k*) within experimental trials 1-3. We have updated the statistical methods to reflect this change at lines 236-239:**

**“****For trials 1-3, linear mixed-effects models (LMM) were constructed to test for sex-specific *p*CO_2_ effects on growth (TL, wW, and *k*) within trials 1-3. To account for a common rearing environment, tank was included as a random effect:**

**TL (wW, *k*) =** ***p*CO_2_ + sex + *p*CO_2_ × sex + tank + error.”**

**In addition to using LMMs for significance testing and Cohen’s d to quantify effect sizes,** **we now also employ a shift function to characterize in more detail how elevated CO_2_ exposure altered not just means, but the trait distributions themselves. The shift function is a powerful tool to explore changes in distribution characteristics. Furthermore, the shift function permits us to quantify CO_2_ effects for each temperature treatment during trial 4, which could not be analyzed via LMM. We describe the shift function methods starting at lines 240-249:**

**“We also analyzed how trait frequency distributions varied between *p*CO_2_ treatments by implementing a series of shift functions [58]. Within each trial (1-4), measurements of TL, wW, and k were pooled from replicate tanks and five quantiles (0.1, 0.25, 0.5, 0.75, and 0.9) from each treatment were computed using a Harrel-Davis quantile estimator [59]. For each trait, quantile estimates from the low *p*CO_2_ treatment were subtracted from the high *p*CO_2_ distribution and 95% confidence intervals (CIs) for quantile differences were calculated using a bootstrap estimation of the standard error of the quantile [60]. Significant CO_2_ effects on quantile differences were assumed if bootstrapped 95% CIs did not include zero. Significance levels for the 5 quantile comparisons were adjusted for multiple comparisons using Hochberg’s method [61].”**

*R1-4: The third major concern is the level of replication applied is very difficult to interpret. As presented is appears that embryos are divided in 3-4 circular vessels in a single main rearing tank per treatment. Then this is adjusted to 3x 50l vessels when larvae reach 10mm, distributed in the rearing vessel, before finally animals are released into the rearing vessel at 1200 degree days. This is 70 days, 50 days and 42 days post hatch respectively for 17, 24 and 28 degrees. So based on an experimental duration of 135 days, 107 days, and 88 days for these temperature treatments respectively, over half the experiment is conducted with all larvae in a single experimental rearing tank per trial. The rearing tank is the level at which the treatment is applied, so this is pseudo-replication, and in the case of the 28 degree treatment there is only 1 replicate. Either this is inadequate study design, or it is inadequate explanation of the exact experimental protocol in the methods. Hopefully this is the latter, and further detail can reassure the editor/reviewers/readers that the level of replication and statistical design was appropriate. Simply including tank, or year, as random effects in a LMM in not sufficient justification, when each year contains a single treatment (thus year would be exactly analogous to treatment effect).*

**We have thoroughly revised the methods section to better describe the level of replication. Trials 1-3 were run using two replicate 700-L tanks per CO_2_ level (four tanks total per trial). At the beginning of each trial, young fish were confined to smaller vessels (20 L and 50 L) nested within the 700-L tanks to maximize the feeding efficacy of larvae and small juveniles on Artemia nauplii. At ~1,200 ddph, juveniles (~25 mm TL) were switched to a powdered diet only and were pooled from the smaller vessels into the same main tank that they developed in. Therefore, two replicate tanks per CO_2_ treatment were maintained for the duration of the trial (see Table 2 for list of tanks used per experiment). However, restrictions on laboratory space limited the trial to N = 1 rearing tank per CO_2_ × temperature treatment. Lines 173-175 now read:**

**“Trials 1-3 were conducted in four 700-L main tanks (N = 2 per CO_2_ treatment). During trial 4, restrictions on laboratory space limited the trial to N = 1 rearing tank per CO_2_ × temperature treatment (Table 2).”**

*R1-5: My final concern is the water chemistry testing. Water used for the experiment came from Long Island sound, and was distributed in the rearing tanks at 300L. Each day 10% of water was changed from each rearing tank. Meaning essentially 100% water change every 10 days (although in reality not quite the case due to dilution). However, during each trial AT and salinity were measured 3 times, at 17 degrees this is 3 times in 135 days. So in the 45 days between AT measurements, water could have change completely 4 times. Given the known dynamics of carbonate chemistry in nearshore coastal regions, parameterising alkalinity this infrequently is not enough to fully describe the carbonate chemistry in this experiment, even if pH is measured daily.*

**We recognize R1’s concern that infrequent sampling for salinity and alkalinity may not reconcile the short-term variation in carbonate chemistry of our seawater source. However, continuous monitoring of our laboratory intake seawater [1] and of an adjacent embayment [2] indicates that local salinity conditions are highly stable, particularly during summer months when these experiments were conducted. For example, during June through October of 2019, the salinity of our laboratory water only varied between 29 and 32 for more than 95% of the period. More than 78% of the measurements varied between 30 and 31.5. While salinity is not a perfect proxy for alkalinity in nearshore environments, we can conclude that changes in alkalinity due to salinity fluctuations would be small. Furthermore, the consistency of within-trial alkalinity measurements further suggested that our seawater chemistry remained stable over the course of the experiment. When combined with our meticulous maintenance of other seawater parameters (including salinity, pH, and nutrients) we are confident that our alkalinity sampling was adequate to estimate experimental *p*CO_2_ levels.**

*R1-6: In the discussion, the authors highlight the issues with empirical data collection, and the requirement of the field to coalesce around a set of standardised methodologies. However, in their own approach, the methodology employed by the authors is certainly far from standardised, optimised or appropriate for the way the data is laid out.* *Undoubtedly this experimental design is a result of constraining factors outside the author’s control (e.g. the requirement to run separate treatments over subsequent years). This alone does not prevent the data being publishable (provided the major issue with the replication can be better explained as shown to be a miscommunication of the real level of replication, which must be far greater). If the data was presented as 3 discrete components/separate experiments (e.g. impact of sex on pCO2 impacts at 17 degrees/ impact of pCO2 on growth at 24 degrees/ impact of growth at 28 degrees), it could add valuable data (albeit far less elegant than the intended aim of this study as initially presented) to the field.*

**We appreciate R1’s constructive recommendations for restructuring our analysis. As described in the previous comments, we have implemented these suggestions and now only report on *p*CO_2_ × sex effects from within trials 1-3. We strongly believe that the inclusion of the shift function has substantially improved our analysis by quantifying changes in trait distributions rather than just measures of central tendency. Furthermore, the shift function allowed to quantitatively analyzing data from trial 4, which could not be analyzed via LMM given the lack of tank replication. Given these substantial analytical improvements, the results section has been completely rewritten. Please refer to lines 262-319 to evaluate the updated results section.**

**Reviewer 2**

*R2-1: I find that the questions asked by the authors to be of contemporary scientific interest and the model species used to be suitable for the questions raised. However, I think that there are certain drawbacks that would require major revisions in the manuscript regarding clarification/reanalyses when it comes to the experimental design and revision of the results that are currently presented in a confusing manner. Please find my specific comments to be detailed below.*

**We thank R2 for her/his comprehensive and constructive evolution of our manuscript. We worked to incorporate her/his suggestions to the best of our ability.**

*R2-2: Title. Please rephrase this to sentence as it is currently quite vague.*

**We excluded the term “temperature-specific” from the title, which now reads “Are long-term growth responses to elevated CO_2_ sex-specific in fish?”**

*R2-3: Line 50. Dated reference, based on what scenario? Latest IPCC projections vary.*

**We recognize that there are more current predictions for long-term atmospheric *p*CO_2_ scenarios. The latest IPCC assessment (AR5) is in agreement with Schmittner et al. (2008). To reflect the more recent assessment, we now cite the AR5 and this sentence has been changed to (line 49-51):**

**“Human activities are rapidly increasing atmospheric and therefore surface ocean carbon dioxide (CO2) [1]. With the unmitigated production of anthropogenic CO2 (i.e., RCP8.5 emissions scenario) these levels could eclipse 2,000 ppmv within the next 300 years [2].”**

*R2-4: Line 52-54. This comes across very dire. Please present negative, neutral and positive effects in the introduction as perhaps see the latest from Jutfelt group (DOI: 10.1038/s41586-019-1903-y).*

**We agree, the full range of responses should be better represented in the introduction. This section now reads (L51-55):**

**“The rapid progression of modern ocean acidification (OA) may challenge physiological tolerance limits of many marine ectotherms [3-5]. In marine fish, responses to future pCO2 conditions have been complex. Experiments have demonstrated a range of positive, neutral, and negative impacts to survival, development, and behavior [6-8].”**

*R2-5* *Line 115: How many of these tanks per treatment? Line 169 suggests to me that everything may be pseudo replicated. One of my main concerns throughout the manuscript is that the trials should be the replicate and within each trial, organisms face the same water (static tanks, correct?) and are therefore pseudo replicates. If I got this wrong, I recommend making this clearer in the methods.*

**We have adjusted the methods section to better describe the level of replication. Trials 1-3 were run using two replicate 700-L tanks per CO_2_ level (four tanks per trial). Offspring were initially confined to smaller vessels (20 L and then 50 L) nested within the 700-L tanks to maximize larval feeding efficiency on Artemia nauplii. At ~1,200 ddph, juveniles (~25 mm TL) were switched to a powdered diet only, and we pooled the juveniles from the smaller vessels into the main tank. However, restrictions on laboratory space limited trial 4 to N = 1 rearing tanks per CO_2_ × temperature treatment. The number of tanks used for each trial is presented in Table 2. Lines 173-175 now read:**

**“****Trials 1-3 were conducted in four 700-L main tanks (N = 2 per CO_2_ treatment). During trial 4****, restrictions on laboratory space limited the trial to N = 1 rearing tank per CO_2_ × temperature treatment (Table 2).”**

**We recognize that the lack of replicate tanks during trial 4 prohibits an analysis by LMM. Instead, we now quantify CO_2_ effects within temperature treatments from trial 4 by using a shift function. The shift function is a powerful tool to explore how two independent distributions change and not just that they differ. We describe the shift function methods starting at lines 240-249:**

**“We also analyzed how trait frequency distributions varied between *p*CO_2_ treatments by implementing a series of shift functions [58]. Within each trial (1-4), measurements of TL, wW, and k were pooled from replicate tanks and five quantiles (0.1, 0.25, 0.5, 0.75, and 0.9) from each treatment were computed using a Harrel-Davis quantile estimator [59]. For each trait, quantile estimates from the low *p*CO_2_ treatment were subtracted from the high *p*CO_2_ distribution and 95% confidence intervals (CIs) for quantile differences were calculated using a bootstrap estimation of the standard error of the quantile [60]. Significant CO_2_ effects on quantile differences were assumed if bootstrapped 95% CIs did not include zero. Significance levels for the 5 quantile comparisons were adjusted for multiple comparisons using Hochberg’s method [61].”**

*R2-6: Line 137: Reference to how long these trials lasted either in table or text here please.*

**The duration of each trial is listed in Table 1. We edited lines 127-128 to highlight this:**

**“A summary of the duration and the conditions applied during each trial is listed in Table 1.”**

*R2-7: Line 195: Discards not sexed?*

**Discards were not sexed.**

*R2-8: Line 196: What were the mortality rates?*

**Early life survival rates were quantified for Trial 1 and they did not vary significantly between CO_2_ treatments (hatch survival: 85%, survival to 10 days post-hatch: 39%). These values have been published previously [4]. We chose not to quantify early-life survival for Trials 2-4 as we have extensively evaluated embryonic and larval effects of CO_2_ across the same temperatures tested here [5]. The early life stage experiments published in Murray & Baumann (2018) were conducted in parallel during the same experimental years as the long-term rearing experiments described in this manuscript.**

*R2-9: Line 214: This needs to be tested.*

**We now report juvenile survival rates for each trial 1-4. We used t-tests to check for significant effects of CO_2_ on survival for trials 1-3, but in all cases, survival was similar between *p*CO_2_ levels within trials.**

*R2-10: Lines 216-217: Were checks made for baseline differences between trials?*

**We are unsure what R2’s meant with “baseline” differences here. We address the differences in final sex ratios observed between trials 1-2 in the results section at lines 259-264 and discussed implications in the second paragraph of the discussion.**

**As we described in the introduction, the sex of Atlantic silversides is determined during the larval stage (between 8 – 21 mm TL) and is partially sensitive to environmental rearing temperature. This is a well-studied phenomenon in this species [6]. We designed our experiments to test for an influence of *p*CO_2_/pH level on sex determination by rearing offspring from fertilization at two *p*CO_2_ levels and under common temperature conditions. Theoretically, offspring from the same fertilization event that are reared at the same temperature should exhibit similar sex ratios, and thus any differences in final sex ratios between *p*CO_2_ treatments could indicate that *p*CO_2_/pH also influences sex determination.**

**There was a small error in the original dataset for sex ratios during trial 1 that has now been corrected. As such, there are no statistical differences in the final sex ratios between *p*CO_2_ levels during trial 1, and sex ratios are very similar between trials 1-2. Please refer to the results section at lines 263-269 for the corrected data and to Fig. 1.**

*R2-11: Lines 225-227: Please revise, confusing.*

**As per the recommendation of both reviewers, we now test for sex-specific growth effects at 17° and 24°C and this section has been deleted. We updated the text at lines 236-239 to reflect this change:**

**“For trials 1-3, linear mixed-effects models (LMM) were constructed to test for sex-specific *p*CO_2_ effects on growth (TL, wW, and *k*).”**

*R2-12: Line 225: Line 217 suggests this was checked at 24C for trial three, so this is confusing. If literature suggests effects occur above 20C, then why test 17C? Why is the interaction of higher temperature and pH not interesting to the authors?*

**The rationale for initially focusing on sex-dependent effects of CO_2_ at 17° but not 24°C was due to the expected differences in female sample sizes. Atlantic silversides from this latitude exhibit temperature-dependent sex determination, which means in practice that rearing ~150 fish at 24°C produces only ~15 females and 135 males. We felt the low female sample size at 24°C was inadequate for a robust statistical comparison. However, after reconsidering we now include a linear mixed-effects model to test for sex-specific effects of CO_2_ on growth despite disparities in sample size.**

*R2-13: Line 230: Seeing the interesting results on Fultons k, I am unsure why wW isn’t included.*

**We agree that omitting wW from statistical testing because TL and wW are correlated is unnecessary. We now test wW using a LMM in same manner as TL and Fulton’s k. Lines 236-249 have been updated to reflect this change in methodology.**

*R2-14: Line 242: The same individual was not compared over time, so a pairwise t-test may not be appropriate.*

**We have adjusted our analysis and now use LMM to test for significant effect of CO_2_ on TL within each subsample group. Tank is included in the model to account for offspring sampled from the same rearing tank. We have updated the methods section at line 250-252:**

**“To evaluate time-dependent effects of elevated *p*CO_2_ exposure, we employed LMMs test for CO_2_ effects on the TL of each group of sub-sampled offspring (Table S2) using the model:**

**TL = *p*CO_2_ + tank + error.”**

*R2-15: Line 247: Prefer a formal stats test for these.*

**We recognize that for some, formal testing of model residuals for normality and variance heterogeneity is preferred. But for large datasets (like those evaluated here) these tests are overly sensitive to small deviations which leads to unnecessary data transformations that can bias results [7]. Therefore, we tested the assumptions of LMMs via visual inspection of residual QQ plots and boxplots. In all cases, our data sufficiently conformed to the required assumptions to proceed with LMMs. We therefore have high confidence that our results are not biased by violations of LMM assumptions.**

*R2-16: Lines 250-256, Line 267: Trials may have to replicate here. They are the source of biological variability in experimental design.*

**Please refer to response R2-5 for a detailed description of the within trial replication.**

*R2-17: Line 270: Ok, but what about sex specific effects?*

**This section has been deleted as we have largely rewritten the results section to incorporate suggestions from both reviewers. Please refer to lines 263-319 for the updated results.**

*R2-18: Line 274-275: Confusing with respect to line 271.*

**This section has been deleted. Please refer to R2-17 for these changes.**

*R2-19: Line 298: Is this essentially the control?*

**Atlantic silversides commonly spawn at temperatures from 17°-24°C and both could be considered control conditions and thus we chose to not explicitly designate a control temperature. The 28°C treatment was designed to represents an anomalously warm reproductive temperature, which occasionally occurs already during peak summer, but may become more frequent in the future.**

*R2-20: Line 307: “co2 effect sizes” which direction?*

**The implementation of Cohen’s d to quantify CO_2_ effects sizes was described in the methods at line 258:**

**“We used Cohen’s d to calculate CO_2_ effect sizes (±95% CIs) using the R package *effsize* [66] where negative values indicate a trait reduction under high *p*CO_2_ [67].”**

**This original sentence has been removed, but we now state the effect size direction where necessary.**

*R2-21: Line 307-308: Or there is simply no effect?*

**This sentence has been deleted, see comment R2-22 below.**

*R2-22: Line 309: Why not no evidence?*
**This original sentence has been deleted. Now that we analyze each trial on its own, we did find that male fish were more affected by high *p*CO_2_ conditions during trial 1. We report these results at lines 272-285. However, this effect was limited to trial 1 and not reproduced in trial 2.**

*R2-23:* *Line 315: Ok, but that life history stage is not tested here either.*

**We feel that it is important to use the discussion to highlight key gaps in knowledge with respect to long-term CO_2_ effects in fish. Our study demonstrated that continuous exposure to elevated *p*CO_2_ – as it is certainly true in future acidified ocean - can lead to detectable growth reductions in juvenile fish. Our findings reject the hypothesis that female fish are more sensitive to high *p*CO_2_, but as we note in the text the energetic requirements of productive maturation tend to be greater in female fish than in males (aside from potential behavioral effects on energetics associated with mating). Therefore, the logical next step for this research is to evaluate how long-term effects of high *p*CO_2_ through reproduction to quantify impacts on reproductive output and offspring fitness. As we also highlight in the text, whole-life cycle effects of high *p*CO_2_ exposure remain woefully understudied.**

*R2-24: Line 319: on what aspects of reprodcutove success?*

**Miller et al. (2015) and Welch et al. (2016) showed how adult CO_2_ exposure modified reproductive output (number of clutches and average clutch size), offspring survival, and offspring growth. We have updated this sentence to be more specific (lines 345-348):**

**“To date, very few studies have quantified CO_2_ impacts on fish reproductive output and offspring viability, reporting inconsistent outcomes [72, 73]”**

*R2-25: Line 322: Not sure if the process of sex determination is the one investigated here.*

**Please see our response to comment to R2-10.**

*R2-26: Line 323: Why only 2000 in line 305 then?*

**The discrepancy in sample sizes between the analyses of sex-specific growth effects and effects on sex-ratios was the inclusion of data from 24°C in the latter. However, we are now including trial 3 data for the analysis of growth effects and thus the sample sizes are the same.**

*R2-27: Line 327-328: Again, to me this evidence suggesting that there are different baslines across trials since they are likely the true replicates. Also, this sentence is quite confusing. Either discuss the 80’s data or don’t but brushing over what is presents seems pointless.*

**Please refer to comment R2-10 for our comment regarding different baselines.**

*R2-28: Lines 322-334: Why not discuss the temperature differences in sex ratio here?*

**Temperature dependent sex determination in *M. menidia* is a well-documented phenomenon that has been examined in great detail. We cited the preeminent studies in the introduction. For example, Duffy et al. (2010) characterized the temperature sensitive gene expression in *M. mendia* during sex differentiation. The scope of this study was to determine if *p*CO_2_ level has an additional effect on sex determination. However, the temperature dependent female sex ratios were largely consistent with previously reported values. To report this, we added a sentence to the discussion at line 331:**

**“The female sex ratios were consistent with previously reported values of ~10% at 24°C and ~45% at 17°C [69].”**

*R2-29: Line 329: What are these sex ratios?*

**This sentence has been deleted. Refer to comment R2-27.**

*R2-30: Line 349-350: To me, it would be interesting if this is in the gonads or where specifically?*

**We share R2’s curiosity for the underlying cause of higher Fulton’s *k* in fish reared at 17°C and under elevated *p*CO_2_ conditions. However, our analysis did not include an evaluation of differential weight accumulation to various tissues between treatments. However, it should be noted that an increase in condition might not be due to an increase in energy storage. In fact, changes in the weight/length ratio (Fulton’s K is one index describing it) are often associated with differences in body shape. We now discuss this point starting at line 382:**

**“Despite their reduced length and weight, we found that juveniles reared at 17°C under acidified conditions consistently exhibited higher Fulton’s *k* values than control conspecifics. Exposure to high *p*CO_2_ conditions led to a greater reduction in average length than weight, hence an increase in Fulton’s *k*. While the basis of this increased condition is unknown, it does suggest that acidified environments change that way in which silversides partition resources. Or, exposure to high *p*CO_2_ changes the shape of developing silversides which would confound comparisons based on conditions factor alone [78]. Atlantic silversides undergo intense size-selective overwintering mortality where large size paired with increased lipid storage is conducive to higher survival [79]. Therefore, a relatively small CO_2_ induced reduction in the size at onset of the overwintering period could have larger implications for Atlantic silverside population dynamics, as smaller fish incur higher winter mortality and produce fewer viable offspring the following spring [80]. An increase in Fulton’s** k **might offset the risk of winter starvation, but this would entirely depend on individuals acclimated to high *p*CO_2_ actively increasing lipid energy stores [81]. In contrast, higher condition values due to changes in shape is not likely to alleviate overwinter mortality. Our understand of the relationship between high *p*CO_2_ exposure and condition factor would benefit from a detailed analysis of energy composition and form factor [82]”**

*R2-31: Line 354: What size is the large size and how much higher survival? How does it compare to your data?*

**Atlantic silversides exhibit hyperallometric scaling with respect to the accumulation of lipid energy reserves [10]. That is, larger and faster growing animals are more likely to put on lipid weight in addition to lean mass, and lipids are the main energy source sustaining silversides during the overwintering period when feeding ceases. However, this is a general pattern and we do not feel it is appropriate to directly compare our data from laboratory-reared fish. Instead, our main point is that if high *p*CO_2_ restricts growth in the wild, then it would be an additional factor reducing overwinter survival.**

*R2-32: Line 355-356: Does this account for higher fultons k observed at higher pCO2?*
**This section has been modified, please refer to comment R2-30.**

*R2-33: Line 358-359: But what if smaller fish produce better quality eggs?*
**In the realm of fish ecology, smaller fish rarely if ever produce better eggs. Instead, fitness in broadcast spawners like the majority of fish species relies on fecundity and repeat spawner experience. In annual species like silversides, it’s solely about fecundity, which scales allometrically with fish size. So no, we cannot rule out that smaller fish produce better quality eggs; but such a relationship is not realistic in silversides. Because Atlantic silversides are an annual species and income batch-spawners, it is thought that their reproductive output is maximized by maximizing body size and ripening the largest number of egg per batch as possible [11]. Years of experimentation on this species showed that there is strong selection for increased body size in female silversides.**

*R2-34: Authors lead discussion along a premise of individuals becoming smaller = negative consequences. I’d like to see some discussion of if there can be a selection towards smakker sied individuals with higher fultons k and what that might imply*

**Please refer to comment R2-30 to see how me now discuss the implications for a higher Fulton’s *k* value.**

*R2-35: Lines 362-363: What concentrations of CO2, what temperature levels? Ie are they comparable? This is too brief, please discuss conflicting data.*

**We have edited this sentence to include the *p*CO_2_ and temperature conditions used to examine early-life stage effects in *M. menidia* (lines 398-400):**

**“Previous work on M. menidia early life stages found growth to be largely unaffected by high *p*CO_2_ conditions (2,000 – 6,000 µatm) across the same range of temperatures examined here (17°-28°C) [48].”**

*R2-36:* *Lines 370-378: These line are repetitive to text in methods/introduction.*

**We agree that this section was redundant with previous sections of the manuscript and it has been deleted. In its place, we now discuss an additional reason why our study found measurable reductions in growth under long-term high *p*CO_2_ exposure. This now reads at lines 406-414:**

**“Another important difference between this and previous long-term experiments was our application of a high *p*CO_2_ treatment of 2,200 µatm. By contrast, most studies that have reported neutral CO_2_ growth responses have exposed fish to ~1,000 *p*CO_2_ [73, 74, 83-86]. While this may highlight the widespread resiliency of fish to predicted end-of-century *p*CO_2_ levels [6], such predictions are generalized for the average global ocean [2]. In contrast, coastal marine systems are already prone to periodic acidification near or in excess of 1,000 *p*CO_2_ [47, 87, 88] and future anthropogenic impacts will likely intensify the duration and magnitude of these events [89, 90]. As such, experimenters should strive to apply *p*CO_2_ treatments that reflect the likely future conditions of the systems where their model organisms live.”**

*R2-37:* *Lines 386-388: without the test, this statement is speculative. Remove.*

**Done.**

*R2-38:* *Lines 394-396: OK, but what about trials 3 and 4?*

**We have updated the fifth paragraph of the discussion to better describe the relationship we observed between CO_2_ effects on growth and rearing temperature. Refer to lines 370-381 in the discussion.**

*R2-39:* *Line 397: How can this be environmentally explained?*

**The emergent consensus, empirically confirmed in at least a few cases, is that variability in nearshore *p*CO_2_ fluctuations results in adaptation to local *p*CO_2_ environments [12, 13]Furthermore, seasonal variation in high *p*CO_2_ tolerance has already been observed in Atlantic silversides [14]. It is plausible that high CO_2_ tolerance varies between adult spawning silversides and their offspring, and therefore outcomes in laboratory experiments may depend on phenotypes of adult spawners. However, this is just one factor that could explain the variance we observed between trials, as covered in this section of the discussion.**

*R2-40:* *Lines 401-403: Obvious, remove. With this in mind, authors should have applied these practices to lines 395-396.*

**While this an obvious point to R2, the lack of standardization in the field has been and remains an obstacle to making informed generalizations about effects of ocean acidification in fish. As in any developing field, experimenters sharing their learned knowledge to improve protocols are valuable contributions to manuscripts. It is also obvious that experimental protocols can be improved through experience, and we would have been remiss to not improve waste management in rearing tanks when the support became available.**

*R2-41:* *Abstract: Please go through the text to standardise text for species names (italics) and pCO2 as in the main manuscript.*

**Done.**

*R2-42:* *Line 38: Not clear what the control temperature treatment is.*

**See comment R2-19.**

*R2-43:* *Line 57: What latitudes/environments?*

**The resiliency of juvenile and adult fish to high CO_2_ has been documented in species across latitudes and environments. It is not necessary to draw a distinction here.**

*R2-44:* *Line 67: Well it’s not just likely, in certain cases, it has been demonstrated. See Mittermayer et al 2019 Scientific Reports for one example.*

**We thank R2 for the citation suggestion. We now cite Mittermayer et al. 2019 to support the idea the high *p*CO_2_ exposure results in differential expression of key regulatory enzymes.**

*R2-45:* *Line 68: too small to detect but what about theoretical calculations?*

**We cite Heuer & Grosell (2016) in the introduction to support the idea that theoretical reductions in growth based on models or measurement on isolated tissues are hard to detect in whole-animals over short timescales. And in fact, the reductions in body size under high *p*CO_2_ observed in this study are of a similar scale to the increased energetic demands observed in isolated tissues as reported by Heuer & Grosell (2016). To better highlight this point in the manuscript, we added the following sentences to the discussion (lines 362-369):**

**“Thus, across trial temperature treatments the average juvenile fish from high *p*CO_2_ conditions was shorter (-2 to -9%) and weighed less (-3 to -18%) than control conspecifics. Intestinal tissues isolated from Gulf toadfish showed an 8% increase in energetic consumption when exposed to 1,900 µatm *p*CO_2_ [26]. The percent reductions in whole-animal size observed here are proportionally similar and likely reflect the increased long-term homeostatic costs of life under high *p*CO_2_.”**

*R2-46:* *Lines 71-73: Again, see recent paper from the Jutfelt group.*

**We thank R2 for this suggestion, but this paper deals entirely with behavior and is thus not the best citation here.**

*R2-47:* *Line 88: How strong is this relationship?*

**In Atlantic silversides this relationship is strong and is believed to have driven the adaptation of temperature-dependent sex determination, thereby allowing females a longer growing season to maximize the size and reproductive fitness the following year [11].**

*R2-48:* *Line 93: “more male biased” This is too vague, please be more specific.*

**For clarity this sentence has been rewritten as (lines 93-95):**

**“While temperature is the most common abiotic cue that controls environmental sex determination in fish [16], in freshwater teleosts exposure to low pH conditions can result in a higher proportion of males in the population [17-19].”**

*R2-49:* *Line 93: “low pH conditions” Please provide the range of these conditions.*

**For the sake of brevity, we believe that it is not necessary list a range of pH conditions here. Particularly because these are freshwater fish, and freshwater systems are characterized by different pH dynamics than marine environments, and are therefore not particularly useful for direct comparison. The rationale for including this information here is to highlight that process of sex determination in teleost fish can be influenced by environmental pH.**

*R2-50:* *Line 94: Specify what the warm conditions are.*

**This sentence has been edited to read (lines 95-99):**

**“In the Atlantic silverside (*Menidia menidia)*, exposure to warm conditions (+17°C) during early larval development (between 8 - 21 mm total length) has a masculinizing effect [20] as warm temperatures suppress the expression of the feminizing enzyme aromatase, thus promoting development of testes [21, 22].”**

*R2-51:* *Line 138: How long were samples stored in these conditions and how?*

**Samples were stored in a dark refrigerator at 3°C for less than two weeks before being analyzed. This sentence has been edited to read (lines 143-145):**

**“Bottles were stored in the dark at 3°C and within two weeks of sampling duplicate measurements of *A*_T_ were made on each seawater sample by endpoint titration (G20 Potentiometric Titrator, Mettler Toledo^®^).”**

*R2-52:* *Line 157: How were they separated by sex?*

**Offspring were not separated by sex. There are no non-invasive ways to determine sex in juveniles of this species. Males and females were reared in common tanks. Sex was only determined by dissection after the fish were euthanized at the end of the trial.**

*R2-53:* *Line 157: What are these low densities? Temperature is a wide range..*

**Fish were kept at <20 individuals in 50 L tanks. The holding temperature reflected the ambient temperature at collection. This sentence has been edited to read (line 161-164):**

**“Adults were transported to the Rankin Seawater Facility (University of Connecticut Avery Point) where they were separated by sex and held for 24-48 h at low densities (<20 fish) in large aerated tanks (50 L, 17°-20°C, ambient *p*CO_2_, no food).”**

*R2-54:* *Line 162: At what ratio? When was fertilisation estimated?*

**Ratios for male and females used for each fertilization are available in Table S1. We did not estimate fertilization success.**

*R2-55:* *Line 176: Specify PAR*

**PAR was not measured.**

*R2-56:* *Line 183: Concentration of shrimp nauplii and small rations?*

**Nauplii and powdered rations were provided *ad libitum.* At this stage, the application of powdered rations is to stimulate first feeding.**

*R2-57:* *Line 185: Time period, concentrations?*

**Rations were provided daily and still *ad libitum.* This sentence has been edited to include *ad libitum*.**

*R2-58:* *Lines 193-194: When, how much?*

**These powdered rations were sprinkled into the rearing tubs at low amounts at the same time the daily nauplii were added. The intent was to acclimatize the fish to feeding on powdered versus nauplii.**

*R2-59:* *Line 230: Change intercorrelated to correlated.*

**This sentence has been removed (see comment R2-13).**

*R2-60: Line 250: Body size date?*

**Date corrected to data.**

*R2-61:* *Line 257: “long term exposure” What is this period exactly?*

**This sentence has been deleted.**

*R2-62:* *Lines 261-262: In which treatments?*

**This section has been deleted and reformatted.**

*R2-63:* *Line 291: Grammar.*

**This sentence has been deleted.**

*R2-64:* *Lines 325-326: Mechanism not studied here, too speculative.*

**This sentence has been deleted.**

*R2-65:* *Line 352-353: Remove.*

**This sentence has been deleted.**

*R2-66: Line 382: What life stage?*

**These studies focused on juvenile and larval fish. This sentence has been edited to read (lines 420-422):**

**“Most short-term studies on juveniles and larvae have found no interaction between ration level and CO_2_ sensitivity [31, 33] including in M. menidia [32], but acidification did exacerbate starvation rates in *Rachycentron canadum* [34].”**

*R2-67: Line 409: Change determination to ratios. Also, check grammar of following sentence.*

**This section has been removed.**

*R2-68: Line 673, Table 1: Why the different number of days within the 24C treatment? Why the different number of tanks?*

**Restrictions on laboratory space limited us to just 4 tanks during the 2017 experimental season. We chose to maximize the utility of that trial by employing a 2 × 2 experimental design, despite only applying a single tank per treatment. Because the data from trial 4 are pseudo replicated, we limit their use to the shift analysis only.**

*R2-69: Line 682, Table 2: Please provide HCO3- data.*

**Data for HCO_3_- would be another derived concentration and given that the table is already at a maximum size to fit on one page, we chose to leave it off.**

*R2-70: Line 691, Table 3: Can ages for tables 1 and 3 be given consistently? Trial 3 looks like it might have significant effects but is not discussed in line 250-256?*

**Ages are now presented as day post-fertilization in both Tables 1 and 3. Despite a trend towards a smaller TL and wW under high CO_2_ during trial 3 (particularly at larger size quantiles) the LMM did not detect a significant effect of CO_2_. However, the shift analysis did reveal important differences between *p*CO_2_ treatments for the larger fish from each distribution. We present these results at line 297-304 and discuss the implications at lines 322-332.**

*R2-71: Line 726, Figure 1: dotted lines not needed as the trend is not studied. Also, for panel 1 (17C), seems higher within each trial. Maybe paired tests here with the trial as the replicate and check for indepedant temperature effects later?*

**We did evaluate how CO_2_ level altered female sex ratios for each trial using a chi-square test. We found that *p*CO_2_ level did not alter sex ratios at 17°C. Please refer to comment R2-10.**

*R2-72: Figure 2: What about trial 3 in table 3?*

**Please note that the original fig. 2 has been removed from the paper and has been replaced by a figure that illustrates frequency distributions and shift functions for TL, wW, and k for trials 1-3.**

*R2-73: Figure 3: Remove dotted lines since same individual TL is not being tracked.*

**Done.**

*R2-74:* *Figure 4: Panel 3, bottom. What is trial 5?*

**This figure has been removed from the manuscript.**

**References Cited in Reply**

1.	Dominion foundation-University of Connecticut Carbon and Oxygen time Series [Internet]. University of Connecticut. 2020 [cited 03-01-2020]. Available from: <https://ducos.uconn.edu/>.

2.	Baumann H. Temperature, pH, DO, and salinity data from Mumford Cove, Connecticut, USA from 2015-2020. Biological and Chemical Oceanography Data Management Office (BCO-DMO). 2020;Dataset version 2020-03-01. doi: 10.1575/1912/bco-dmo.660079.

3.	Schmittner A, Oschlies A, Matthews HD, Galbraith ED. Future changes in climate, ocean circulation, ecosystems, and biogeochemical cycling simulated for a business‐as‐usual CO_2_ emission scenario until year 4000 AD. Global biogeochemical cycles. 2008;22(1).

4.	Murray CS, Fuiman LA, Baumann H. Consequences of elevated CO_2_ exposure across multiple life stages in a coastal forage fish. ICES Journal of Marine Science. 2017;74(4):1051-61. doi: 10.1093/icesjms/fsw179.

5.	Murray CS, Baumann H. You better repeat it: complex CO_2_ × temperature effects in Atlantic silverside offspring revealed by serial experimentation. Diversity. 2018;10(3):69. doi: 10.3390/d10030069.

6.	Conover DO, Kynard BE. Environmental sex determination: interaction of temperature and genotype in a fish. Science. 1981;213:31.

7.	Zuur AF, Ieno EN, Elphick CS. A protocol for data exploration to avoid common statistical problems. Methods in Ecology and Evolution. 2010;1(1):3-14. doi: 10.1111/j.2041-210X.2009.00001.x.

8.	Welch MJ, Munday PL. Contrasting effects of ocean acidification on reproduction in reef fishes. Coral Reefs. 2016;35(2):485-93. doi: 10.1007/s00338-015-1385-9.

9.	Miller GM, Kroon FJ, Metcalfe S, Munday P. Temperature is the evil twin: effects of increased temperature and ocean acidification on reproduction in a reef fish. Ecological Applications. 2015;25(3):603-20.

10.	Schultz ET, Conover DO. Latitudinal differences in somatic energy storage: adaptive responses to seasonality in an estuarine fish (Atherinidae: *Menidia menidia*). Oecologia. 1997;109(4):516-29.

11.	Conover DO. Adaptive significance of temperature-dependent sex determination in a fish. The American Naturalist. 1984;123(3):297-313.

12.	Vargas CA, Lagos NA, Lardies MA, Duarte C, Manríquez PH, Aguilera VM, et al. Species-specific responses to ocean acidification should account for local adaptation and adaptive plasticity. Nature Ecology & Evolution. 2017;1:0084.

13.	Hofmann G, Evans T, Kelly M, Padilla-Gamiño J, Blanchette C, Washburn L, et al. Exploring local adaptation and the ocean acidification seascape–studies in the California Current Large Marine Ecosystem. Biogeosciences. 2014;11(4):1053-64.

14.	Murray CS, Malvezzi A, Gobler CJ, Baumann H. Offspring sensitivity to ocean acidification changes seasonally in a coastal marine fish. Marine Ecology Progress Series. 2014;504:1-11. doi: 10.3354/meps10791.

15.	Heuer RM, Grosell M. Elevated CO_2_ increases energetic cost and ion movement in the marine fish intestine. Scientific Reports. 2016;6:34480. doi: 10.1038/srep34480.

16.	Baroiller JF, D’Cotta H, Saillant E. Environmental effects on fish sex determination and differentiation. Sexual Development. 2009;3(2-3):118-35. doi: 10.1159/000223077.

17.	Römer U, Beisenherz W. Environmental determination of sex in Apistogrammai (Cichlidae) and two other freshwater fishes (Teleostei). Journal of Fish Biology. 1996;48(4):714-25.

18.	Rubin DA. Effect of pH on sex ratio in cichlids and a poecilliid (Teleostei). Copeia. 1985;1985(1):233-5.

19.	Oldfield RG. Genetic, abiotic and social influences on sex differentiation in cichlid fishes and the evolution of sequential hermaphroditism. Fish and Fisheries. 2005;6(2):93-110.

20.	Conover DO, Heins SW. Adaptive variation in environmental and genetic sex determination in a fish. Nature. 1987;326(6112):496-8. doi: 10.1038/326496a0.

21.	Penman DJ, Piferrer F. Fish gonadogenesis. Part I: genetic and environmental mechanisms of sex determination. Reviews in Fisheries Science. 2008;16(sup1):16-34. doi: 10.1080/10641260802324610.

22.	Duffy TA, Picha ME, Won ET, Borski RJ, McElroy AE, Conover DO. Ontogenesis of gonadal aromatase gene expression in Atlantic silverside (*Menidia menidia*) populations with genetic and temperature-dependent sex determination. Journal of Experimental Zoology Part A: Ecological Genetics and Physiology. 2010;313A(7):421-31. doi: 10.1002/jez.612.
